# Anti-Inflammatory Effects of *Mitrephora sirikitiae* Leaf Extract and Isolated Lignans in RAW 264.7 Cells

**DOI:** 10.3390/molecules27103313

**Published:** 2022-05-21

**Authors:** Supachoke Mangmool, Chayaporn Limpichai, Khine Kyi Han, Vichai Reutrakul, Natthinee Anantachoke

**Affiliations:** 1 Department of Pharmacology, Faculty of Science, Mahidol University, Bangkok 10400, Thailand; supachoke.man@mahidol.ac.th; 2 Department of Pharmacognosy, Faculty of Pharmacy, Mahidol University, Bangkok 10400, Thailand; chayapornlim@gmail.com; 3 Department of Pharmacology, University of Pharmacy, Yangon 11031, Myanmar; khinekyihanuop@gmail.com; 4 Department of Chemistry, Faculty of Science, Mahidol University, Bangkok 10400, Thailand; vichai.reu@mahidol.ac.th; 5 Center of Excellence for Innovation in Chemistry, Faculty of Science, Mahidol University, Bangkok 10400, Thailand

**Keywords:** *Mitrephora sirikitiae*, anti-inflammation, lignans, polyphenols, mRNA expression, cytokines

## Abstract

*Mitrephora sirikitiae* Weeras., Chalermglin & R.M.K. Saunders has been reported as a rich source of lignans that contribute to biological activities and health benefits. However, cellular anti-inflammatory effects of *M. sirikitiae* leaves and their lignan compounds have not been fully elucidated. Therefore, this study aimed to investigate the anti-inflammatory activities of methanol extract of *M. sirikitiae* leaves and their lignan constituents on lipopolysaccharide (LPS)-induced inflammation in RAW 264.7 mouse macrophage cells. Treatment of RAW 264.7 cells with the methanol extract of *M. sirikitiae* leaves and its isolated lignans, including (−)-phylligenin (**2**) and 3′,4-*O*-dimethylcedrusin (**6**) significantly decreased LPS-induced prostaglandin E_2_ (PGE_2_) and nitric oxide (NO) productions. These inhibitory effects of the extract and isolated lignans on LPS-induced upregulation of PGE_2_ and NO productions were derived from the suppression of cyclooxygenase 2 (COX-2) and inducible nitric oxide synthase (iNOS) production, respectively. In addition, treatment with 2-(3,4-dimethoxyphenyl)-6-(3,5-dimethoxyphenyl)-3,7-dioxabicyclo[3.3.0]octane (**3**) and mitrephoran (**5**) was able to suppress LPS-induced tumor necrosis factor alpha (TNF-α) secretion and synthesis in RAW 264.7 cells. These results demonstrated that *M. sirikitiae* leaves and some isolated lignans exhibited potent anti-inflammatory activity through the inhibition of secretion and synthesis of PGE_2_, NO, and TNF-α.

## 1. Introduction

Inflammation is a defensive mechanism that responds to foreign substances or pathogens. During inflammatory reactions, prostaglandin E_2_ (PGE_2_) and nitric oxide (NO) are the crucial proinflammatory mediators. Cyclooxygenase 2 (COX-2) and inducible nitric oxide synthase (iNOS), the key enzymes responsible for the production of inflammatory PGE_2_ and NO, have been identified in activated macrophages [1,2,3]. Lipopolysaccharide (LPS; a pathogen- and host-derived molecule) stimulates macrophages to, in turn, upregulate inflammatory mediators such as PGE_2_, tumor necrosis factor alpha (TNF-α), proinflammatory cytokines (e.g., interleukin (IL)-1, IL-6, IL-8, and IL-10), reactive oxygen species (ROS), and NO [1,4,5,6]. Upregulation of various types of inflammatory mediators leads to acute and chronic inflammation, which is associated with many chronic diseases such as gout, arthritis, diabetes, cancer, atherosclerosis, and neurodegenerative disease [1,2,7,8]. Therefore, inhibition of secretion and synthesis of these inflammatory mediators is a potential therapeutic treatment of inflammation-associated diseases.

*Mitrephora sirikitiae* Weeras., Chalermglin & R.M.K. Saunders (Annonaceae), an endemic plant called Mahaphrom Rachini in Thai, was found for the first time in the Mae Surin Waterfall National Park, Mae Hong Sorn Province, Thailand in 2004 [9]. Recently, we reported anticancer activities of the methanol extracts of *M. sirikitiae* leaves and stems, as well as their isolated compounds (e.g., lignans, dihydrobenzofuran lignan, alkaloids, and diterpenoids) against various types of cancer cells. It was found that lignans, including (−)-epieudesmin (**1**), (−)-phylligenin (**2**), 2-(3,4-dimethoxyphenyl)-6-(3,5-dimethoxyphenyl)-3,7-dioxabicyclo[3.3.0]octane (**3**), magnone A (**4**), mitrephoran (**5**), and 3′,4-*O*-dimethylcedrusin (**6**), were the main secondary metabolites in *M. sirikitiae* leaves [10]. Lignans are polyphenols that possess various pharmacological activities such as antioxidant, anti-inflammatory, antimicrobial, and anticancer activities [11,12,13]. Lignan-rich plant extracts and their lignans have been revealed for anti-inflammatory activities through inhibition of 15-lipooxygenase (15-LOX), COX-1, and COX-2 activities [14] and suppression of PGE_2_, NO, TNF-α, and IL-6 secretions [15,16,17,18,19], as well as downregulation of COX-2, iNOS, TNF-α, and IL-6 mRNA expression [15,16].

However, the anti-inflammatory properties of *M. sirikitiae* leaf extract are not fully understood. The cellular anti-inflammatory activities of the extract and isolated lignans from *M. sirikitiae* leaves on inhibition of LPS-induced upregulation of inflammatory mediators have not been defined. Therefore, we investigated the anti-inflammatory effects of methanol extract of *M. sirikitiae* leaves and its isolated lignans in LPS-induced secretion and synthesis of inflammatory mediators in RAW 264.7 macrophages.

## 2. Results and Discussion

### 2.1. Effects of the Methanol Extract and Isolated Lignans on Cytotoxicity

**Figure 1:**
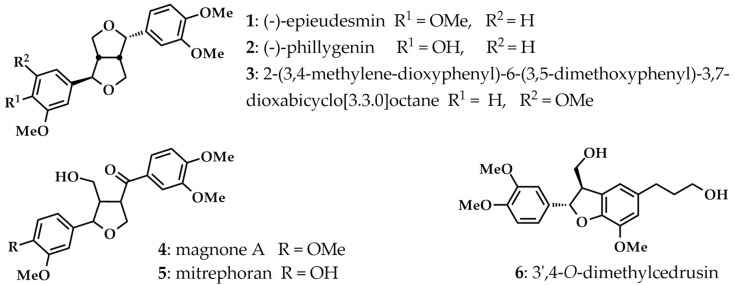
Isolated lignans **1**–**6** from the methanol extract of *M. sirikitiae* leaves.

Cytotoxicity of the methanol extract and isolated lignans from *M. sirikitiae* leaves, including (−)-epieudesmin (**1**), (−)-phylligenin (**2**), 2-(3,4-dimethoxyphenyl)-6-(3,5-dimethoxyphenyl)-3,7-dioxabicyclo[3.3.0]octane (**3**), magnone A (**4**), mitrephoran (**5**), 3′,4-*O*-dimethylcedrusin (**6**) (Figure 1), was evaluated in RAW 264.7 cells using MTT colorimetric assay in order to assign the optimal concentrations of the samples before investigating anti-inflammatory activities in the cells.

**Figure 2:**
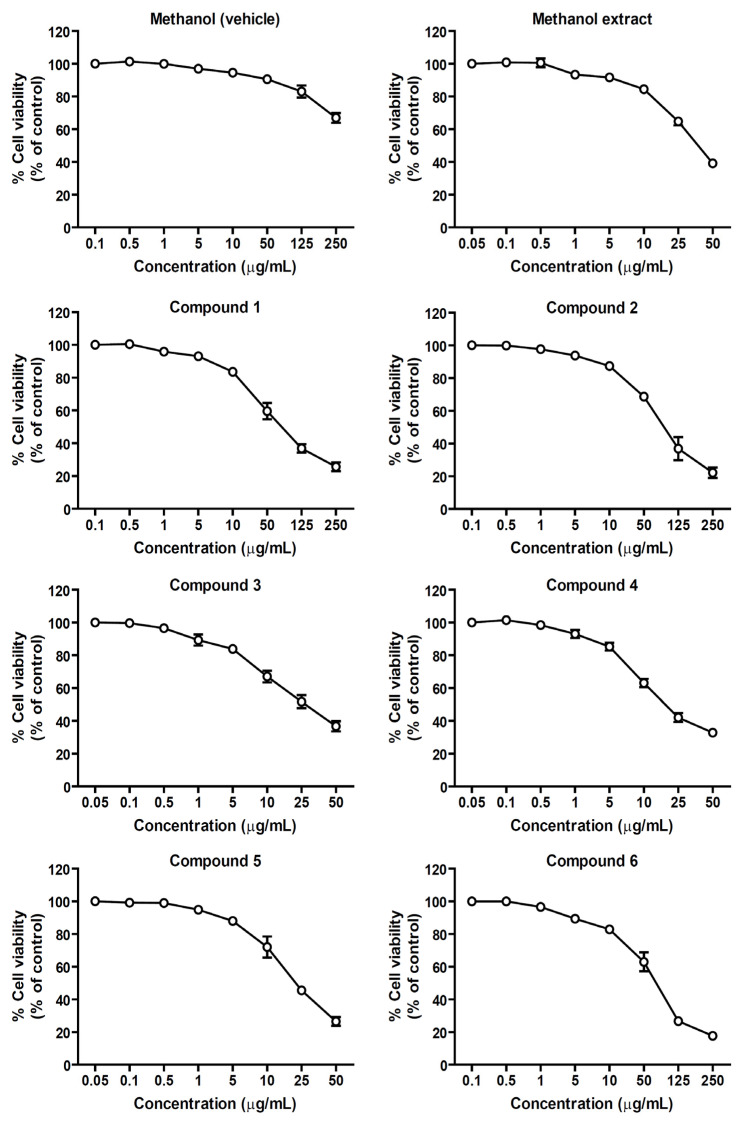
Cytotoxic effects of the methanol extract and isolated lignans **1**–**6** from *M. sirikitiae* leaves in RAW 264.7 cells; The percentage of cell viability of RAW 264.7 cells exposed to 0.05–50 µg/mL of methanol extract and 0.1–250 µg/mL of isolated lignans **1**–**6**. Data are shown as the mean ± SEM (*n* = 5).

After treatment of the cells with various concentrations of the samples for 24 h, the number of viable cells was measured and calculated as the percentage of viable cells compared to a nontreated (control) group. As shown in Figure 2, the MTT results revealed that the number of survival cells more than 80% were found in the groups treated with 0.05–10 µg/mL of the methanol extract, lignans **1**, **2**, or **6**, and 0.05–5 µg/mL of lignans **3**, **4**, or **5**. In order to avoid the cytotoxic effect and allow at least 80% cell viability, the suitable sample concentrations that were used for determining anti-inflammatory effects were 10 µg/mL for the methanol extract, lignans **1**, **2**, and **6**, and 5 µg/mL for lignans **3**, **4**, and **5**.

### 2.2. Effects of the methanol Extract and Isolated Lignans on LPS-Induced PGE_2_ and TNF-α Secretion

**Figure 3:**
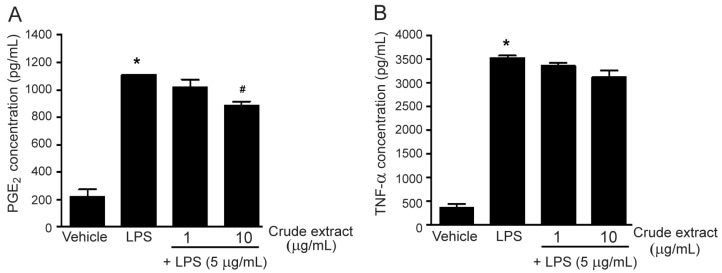
Effects of the methanol extract of *M. sirikitiae* leaves on LPS-induced PGE_2_ and TNF-α secretion in RAW 264.7 cells; Serum-starved cells were pretreated with various concentrations of 0, 1, and 10 µg/mL of crude methanol extract for 3 h, and then stimulated with LPS (5 µg/mL) for 24 h at 37 °C. The levels of PGE_2_ and TNF-α secreted into the medium were assessed by ELISA assay. The PGE_2_ (**A**) and TNF-α (**B**) levels were quantified using a standard curve and expressed as the mean ± SEM (*n* = 3). *, *p* < 0.05 vs. vehicle; #, *p* < 0.05 vs. LPS.

Lipopolysaccharide (LPS), known as a major factor involved in inflammation, is a component of the outer membrane of Gram-negative bacteria. Moreover, it is called endotoxin because of its endotoxic properties. LPS contributes to the pathogenicity of bacteria and can activate several immune cells, including macrophages [20]. In this study, LPS was used for inducing inflammation in RAW 264.7 macrophages. Incubation of the cells with LPS (5 µg/mL) for 24 h markedly increased PGE_2_ and TNF-α secretion (Figure 3). The methanol extract of *M. sirikitiae* leaves was first assessed for the inhibitory effects on LPS-induced PGE_2_ and TNF-α secretions. Treatment of the cells with the methanol extract of *M. sirikitiae* leaves resulted in a decrease in LPS-induced PGE_2_ secretion in a dose-dependent manner, and the maximal inhibitory effect of the methanol extract was observed at a concentration of 10 µg/mL (Figure 3A). However, the methanol extract exhibited little effect on inhibition of LPS-induced TNF-α secretion at the same concentration and did not inhibit LPS-induced TNF-α secretion at a concentration of 1 µg/mL (Figure 3B). These results indicated that the compounds found in *M. sirikitiae* leaves might have anti-inflammatory effects by reducing the secretion of PGE_2_ in RAW 264.7 macrophages.

**Figure 4:**
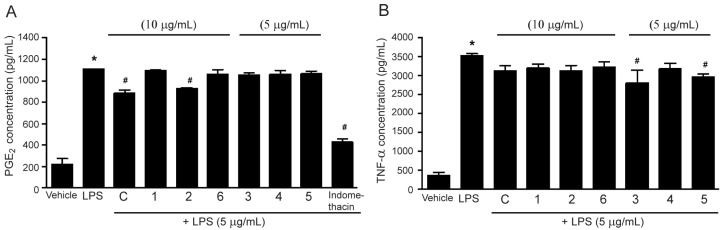
Effects of the methanol extract and isolated lignans **1**–**6** from *M. sirikitiae* leaves on LPS-induced PGE_2_ and TNF-α secretion in RAW 264.7 cells; Serum-starved cells were pretreated with crude methanol extract (C) (10 µg/mL), lignans (5 or 10 µg/mL), or indomethacin (10 µM) for 3 h, and then stimulated with LPS (5 µg/mL) for 24 h at 37 °C. The levels of PGE_2_ and TNF-α secreted into the medium were assessed by ELISA assay. The relative PGE_2_ (**A**) and TNF-α (**B**) levels were quantified using a standard curve and expressed as the mean ± SEM (*n* = 3). *, *p* < 0.05 vs. vehicle; #, *p* < 0.05 vs. LPS.

Lignans are polyphenols, and the main components of *M. sirikitiae* leaves. Our previous study has revealed that lignans (−)-epieudesmin (**1**), (−)-phylligenin (**2**), 2-(3,4-dimethoxyphenyl)-6-(3,5-dimethoxyphenyl)-3,7-dioxabicyclo[3.3.0]octane (**3**), magnone A (**4**), mitrephoran (**5**), and 3′,4-*O*-dimethylcedrusin (**6**) are the main secondary metabolites in *M. sirikitiae* leaves [10]. As shown in Figure 4A, treatment of the cells with (−)-phylligenin (**2**) (10 µg/mL) significantly inhibited LPS-induced PGE_2_ secretion. However, (−)-epieudesmin (**1**), 2-(3,4-dimethoxyphenyl)-6-(3,5-dimethoxyphenyl)-3,7-dioxabicyclo[3.3.0]octane (**3**), magnone A (**4**), mitrephoran (**5**), and 3′,4-*O*-dimethylcedrusin (**6**) had no effect on the inhibition of LPS-induced PGE_2_ secretion. These results are consistent with previous studies, which reported that phylligenin and koreanaside A, lignans isolated from *Forsythia koreana* fruits and flowers, could inhibit PGE_2_ secretion in LPS-stimulated RAW 264.7 cells in a dose-dependent manner [15,16]. Moreover, the anti-inflammation of koreanaside A was also represented by the inhibitory effects on NO, TNF-α, and IL-6 productions [16].

Interestingly, all lignans isolated from the caulis of *Urceola rosea* exhibited anti-inflammatory activities by suppressing LPS-induced synthesis of NO, TNF-α, and/or IL-6 in RAW 264.7 cells. In addition, among those lignans, ecdysanol A and ecdysanol F exhibited potent anti-inflammatory activity against TNF-α secretion with IC_50_ values of 22.9 and 41.9 µM, respectively [17]. Consistent with this previous study, our results showed that among those six lignans isolated from *M. sirikitiae* leaves, 2-(3,4-dimethoxyphenyl)-6-(3,5-dimethoxyphenyl)-3,7-dioxabicyclo[3.3.0]octane (**3**) and mitrephoran (**5**) (5 µg/mL) significantly inhibited LPS-induced TNF-α secretion in RAW 264.7 cells (Figure 4B).

### 2.3. Effects of the Methanol Extract and Isolated Lignans on LPS-Induced Nitric Oxide Production

**Figure 5:**
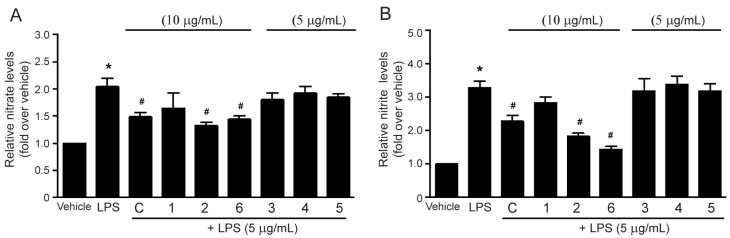
Effects of the methanol extract and isolated lignans **1**–**6** from *M. sirikitiae* leaves on LPS-induced nitrate and nitrite productions in RAW 264.7 cells; Serum-starved cells were pretreated with crude methanol extract (C) (10 µg/mL) or lignans (5 or 10 µg/mL) for 3 h, and then stimulated with LPS (5 µg/mL) for 24 h at 37 °C. The levels of nitrate and nitrite in the medium were assessed by a total nitric oxide assay. The relative nitrate (**A**) and nitrite (**B**) productions were quantified using a standard curve and expressed as the mean ± SEM (*n* = 3). *, *p* < 0.05 vs. vehicle; #, *p* < 0.05 vs. LPS.

Nitric oxide (NO) is a free-radical signaling molecule playing a role in many biological processes, including inflammation. NO is synthesized from L-arginine and oxygen by using NOS as catalysts. There are three main isoforms of NOS, endothelial NOS (eNOS), neuronal NOS (nNOS), and inducible NOS (iNOS). The iNOS enzyme is a major isoform in the inflammatory process [3,21,22]. Thus, upregulation of iNOS might be reflected in NO production in the cells during inflammation. NO is immediately oxidized to generate nitrate and nitrite, both of which are used for measuring NO levels in the cells. Therefore, this study investigated the inhibitory effects of methanol extract and isolated lignans from *M. sirikitiae* leaves on LPS-mediated NO production by determining the levels of nitrate and nitrite in RAW 264.7 cells. Incubation of the cells with LPS (10 µg/mL) robustly increased nitrate and nitrite productions (Figure 5). Treatment with the methanol extract, (−)-epieudesmin (**1**), (−)-phylligenin (**2**), or 3′,4-*O*-dimethylcedrusin (**6**) at a concentration of 10 µg/mL resulted in a significant reduction of LPS-induced nitrate and nitrite levels in RAW 264.7 cells (Figure 5A and Figure 5B, respectively). In contrast, treatment with 2-(3,4-dimethoxyphenyl)-6-(3,5-dimethoxyphenyl)-3,7-dioxabicyclo[3.3.0]octane (**3**), magnone A (**4**), and mitrephoran (**5**) (5 µg/mL) had no effect (Figure 5). These results indicated that the lignans found in *M. sirikitiae* leaves, including (−)-epieudesmin (**1**), (−)-phylligenin (**2**), and 3′,4-*O*-dimethylcedrusin (**6**) exhibited anti-inflammatory activities by attenuating NO production in RAW 264.7 cells. Consistent with our present data, many previous studies have demonstrated that some lignans can suppress NO production during inflammation. For example, the methanol extract of *F. koreana* fruits and its isolated lignan, phylligenin, could inhibit NO synthesis in LPS-treated RAW 264.7 cells [15]. Furthermore, nine lignans isolated *Acanthopanax sessiliflorus* fruits [18] and two dimeric lignans isolated from *Zanthoxylum podocarpum* barks [19] have been reported to have inhibitory effects on NO production in LPS-treated RAW 264.7 macrophages. Thus, natural phenols found in plants as lignans may serve as potential anti-inflammatory agents via inhibition of the NO signaling pathway.

### 2.4. Effects of the Methanol Extract and Isolated Lignans on LPS-Induced mRNA Expressions of Inflammatory Biomarkers

**Figure 6:**
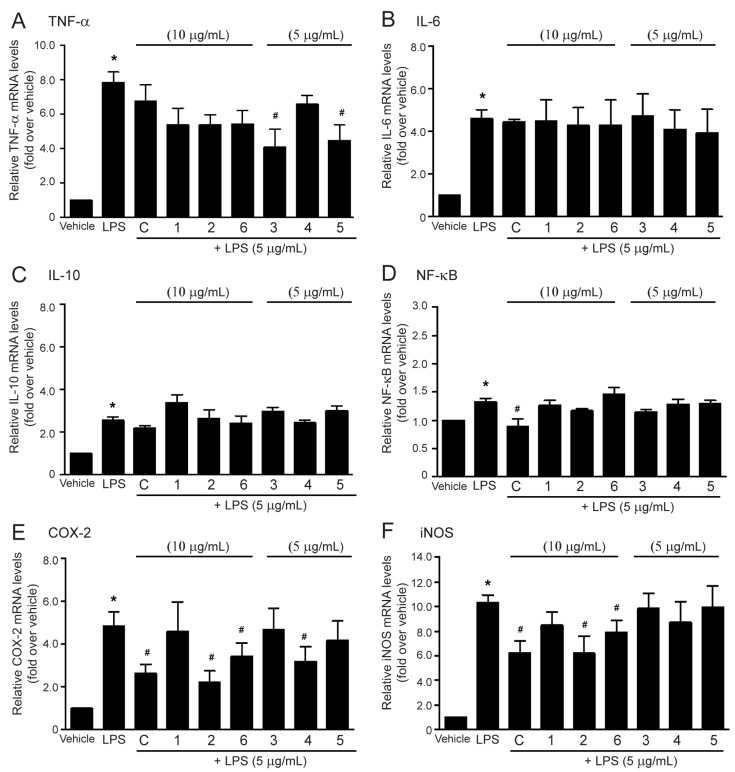
Effects of the methanol extract and isolated lignans **1**–**6** from *M. sirikitiae* leaves on LPS-induced mRNA expressions of inflammatory biomarkers in RAW 264.7 cells; Serum-starved cells were pretreated with crude methanol extract (C) (10 µg/mL) or lignans (5 or 10 µg/mL) for 3 h, and then stimulated with LPS for 6 h at 37 °C. After treatment, the total RNA was extracted from the cells and the mRNA expressions of TNF-α (**A**), IL-6 (**B**), IL-10 (**C**), NF-κB (**D**), COX-2 (**E**), and iNOS (**F**) were analyzed by RT-qPCR with gene-specific primers. The relative mRNA levels were quantified and shown as the mean ± SEM (*n* = 3). *, *p* < 0.05 vs. vehicle; #, *p* < 0.05 vs. LPS.

According to the methanol extract of *M. sirikitiae* leaves and some isolated lignans representing the inhibitory effect on LPS-mediated PGE_2_, TNF-α, and nitric oxide secretions in RAW 264.7 cells, the mRNA expression of inflammatory biomarkers, including COX-2, iNOS, TNF-α, IL-6, IL-10, and NF-κB affected by the methanol extract and isolated lignans **1**–**6** was subsequently studied. In this study, incubation of the RAW 264.7 cells with LPS (5 µg/mL) markedly increased TNF-α, IL-6, IL-10, NF-κB, COX-2, and iNOS mRNA expression levels as compared with a control group (Figure 6A, Figure 6B, Figure 6C, Figure 6D, Figure 6E, and Figure 6F, respectively), indicating the induction of inflammation. The methanol extract of *M. sirikitiae* leaves was able to suppress LPS-induced mRNA expression of NF-κB, COX-2, and iNOS (Figure 6D, Figure 6E, and Figure 6F, respectively) while treatment of the cells with this methanol extract tended to inhibit LPS-induced TNF-α and IL-10 syntheses (Figure 6A and Figure 6C, respectively). However, the extract did not affect IL-6 mRNA expression (Figure 6B). These results demonstrated that the methanol extract of *M. sirikitiae* leaves possesses anti-inflammatory effects by suppressing the synthesis of several inflammatory mediators, indicating that the anti-inflammatory effects are derived from some active compounds contained in *M. sirikitiae* leaves.

Moreover, all isolated lignans **1**–**6** were investigated for their effects on suppressing mRNA expressions of inflammatory mediators induced by LPS. As shown in Figure 6A, treatment of the cells with (−)-epieudesmin (**1**), (−)-phylligenin (**2**), magnone A (**4**), and 3′,4-*O*-dimethylcedrusin (**6**) tended to inhibit LPS-induced mRNA expression of TNF-α. In addition, treatment of the cells with 2-(3,4-dimethoxyphenyl)-6-(3,5-dimethoxyphenyl)-3,7-dioxabicyclo[3.3.0]octane (**3**) and mitrephoran (**5**) significantly inhibited LPS-induced TNF-α mRNA expression. Macrophage cells are the main source of proinflammatory cytokine TNF-α, which is used as one of the major markers of inflammation. This study reported for the first time that isolated lignans **3** and **5** from *M. sirikitiae* leaves exhibited the anti-inflammatory effects by suppressing LPS-induced TNF-α secretion (Figure 4B) and synthesis (Figure 6A) in RAW 264.7 cells.

However, lignans **1**–**6** did not show the inhibitory effects on LPS-induced mRNA expression of inflammatory cytokines, IL-6, IL-10, and NF-κB in RAW 264.7 cells (Figure 6B–D). After exposure to a pathogen (e.g., LPS), macrophages are activated to produce pro-inflammatory cytokines such as IL-1 and IL-6, which play an important role in the inflammatory response [23]. Although the lignans isolated from *M. sirikitiae* leaves did not show the inhibitory effects on LPS-induced IL synthesis, the previous study demonstrated that other types of lignans isolated from the caulis of *U. rosea* exhibited anti-inflammatory effects by reducing the LPS-induced IL-6 production in RAW 264.7 cells with the IC_50_ values ranging from 5 to 50 μM [17]. Thus, each lignan has its own anti-inflammatory properties and mechanisms.

NF-κB is one of the major regulators of inflammatory gene expression. The activation of NF-κB resulted in the upregulation of mRNA expression of cytokines such as TNF-α, IL-1*β*, IL-6, and IL-8, including COX-2 [24]. Even though the methanol extract of *M. sirikitiae* leaves was able to suppress LPS-induced NF-κB synthesis, all lignans isolated from the *M. sirikitiae* leaves had no effect on the inhibition of NF-κB synthesis induced by LPS (Figure 6D). Further studies are required to identify other constituents that have inhibitory effects on the synthesis of NF-κB.

Interestingly, treatment with (−)-phylligenin (**2**), magnone A (**4**), and 3′,4-*O*-dimethylcedrusin (**6**) significantly inhibited the mRNA expression of COX-2 in LPS-stimulated RAW 264.7 cells (Figure 6E). The effects of the methanol extract and (−)-phylligenin (**2**) on COX-2 mRNA expression were correlated with their inhibitory effects on LPS-induced PGE_2_ secretion in RAW 264.7 cells. Our data are in concordance with other studies that some lignans, such as koreanaside A isolated from *F. koreana* flowers, could downregulate COX-2 mRNA expression and attenuate PGE_2_ secretion in LPS-induced RAW 264.7 cells [16]. The methanol extracts of *Schisandra rubriflora* and *Schisandra chinensis* and their isolated lignans, including 6-obenzoylgomisin O, schisandrin, gomisin D, gomisin N, and schisantherin A, have been revealed to exhibit significant COX-2 inhibitory activity [14]. Our study demonstrated the inhibitory effects of magnone A (**4**), and 3′,4-*O*-dimethylcedrusin (**6**) on LPS-induced COX-2 synthesis for the first time. During inflammation, arachidonic acid converses into PGE_2_ mediated through COX-2 catalytic reaction. Inhibition of COX-2 activity and synthesis has been proposed as a useful treatment for various inflammatory diseases [25]. Therefore, COX-2 is a well-known target of various anti-inflammatory drugs such as aspirin and other nonsteroidal anti-inflammatory drugs (NSAIDs). In our present study, the methanol extract of *M. sirikitiae* leaves could inhibit the synthesis of COX-2 and reduce PGE_2_ secretion in LPS-treated macrophages. Thus, *M. sirikitiae* might be used as a medicinal plant for the prevention and restoration of inflammatory diseases.

The iNOS is an inducible isoform of NOS and upregulation of iNOS activity and synthesis occurred in response to inflammation [22]. Therefore, the inhibitory effect on iNOS enzyme, which subsequently suppresses NO generation was investigated. We found that treatments of the cells with the methanol extract of *M. sirikitiae* leaves, (−)-phylligenin (**2**), and 3′,4-*O*-dimethylcedrusin (**6**) significantly inhibited LPS-induced iNOS mRNA expression in RAW 264.7 macrophages, while (−)-epieudesmin (**1**), 2-(3,4-dimethoxyphenyl)-6-(3,5-dimethoxyphenyl)-3,7-dioxabicyclo[3.3.0]octane (**3**), magnone A (**4**), and mitrephoran (**5**) had no effects (Figure 6F). These inhibitory effects of the methanol extract, together with (−)-phylligenin (**2**), and 3′,4-*O*-dimethylcedrusin (**6**) on iNOS mRNA expression (Figure 5F) were correlated with their inhibitory effects on LPS-induced NO production (Figure 4) in RAW 264.7 cells. Interestingly, phylligenin (10–100 µM) and koreanaside A (20–80 µM) isolated from *F. koreana* have previously reported the inhibitory effects on LPS-induced iNOS synthesis in RAW 264.7 cells [15,16]. These findings from ours and previous studies confirmed the anti-inflammatory effects of (−)-phylligenin (**2**) by inhibiting iNOS synthesis and subsequent reducing NO production. Moreover, our study reported the inhibitory effects on iNOS synthesis of 3′,4-*O*-dimethylcedrusin (**6**), the lignan isolated from *M. sirikitiae* leaves, for the first time.

In this study, the structure–activity relationships (SARs) of the natural lignans for anti-inflammatory properties were considered in order to identify the specific structures and functional groups that play an important role in the activities. Based on a variety of chemical core skeletons and functional groups of lignans, isolated lignans **1**–**6** from *M. sirikitiae* leaves could be classified into three subgroups, including furofurans (lignans **1**–**3**), furans (lignans **4**–**5**), and benzofurans (lignan **6**) [26]. The obtained results from our present study indicated that the possible SARs of the anti-inflammatory lignans are different in each specific mechanism of action. For instance, substitution of a hydroxyl group on the phenyl ring and a furofuran moiety as found in lignan **2** might be important for the inhibitory effects against LPS-induced PGE_2_ secretion as well as COX-2 mRNA expression. Moreover, the furofuran with hydroxy-substituted phenyl (lignan **2**) and benzofuran (lignan **6**) skeletons played a determinant role against NO production and iNOS mRNA expression induced by LPS in cells. Meanwhile, furofuran with non-hydroxy-substituted phenyl (lignan **3**) and furan with hydroxy-substituted phenyl (lignan **5**) moieties were found to be essential for the inhibition of LPS-induced TNF-α secretion and TNF-α mRNA expression. Although the SARs of lignans for the downregulation of LPS-induced mRNA expression of IL-6, IL-10, and NF-κB could not be discussed in this study, some previous studies have disclosed that furofuran skeleton is possibly related to the suppression of NF-κB signaling pathway [27]. Moreover, the SARs of coumarinolignans have reported that the substitution of hydroxyl groups on the phenyl ring is important to anti-inflammatory activity [28]. In order to fully understand SARs of lignans for anti-inflammatory properties, a number of lignans with different skeletal types and functional groups are required for the biological testing.

It should be noted that our data were obtained from LPS-induced inflammation in RAW 264.7 cells, and therefore may not reflect the actual systemic anti-inflammatory effects in the human body. Thus, further studies will be necessary to evaluate the anti-inflammatory activities in vivo studies.

## 3. Materials and Methods

### 3.1. Chemicals and Reagents

Lipopolysaccharide (LPS from *Salmonella enterica* serotype Typhimurium), indomethacin, and 3-[4,5-dimethylthiazol-2-yl]-2,5-diphenyltetrazolium bromide (MTT) were purchased from Sigma-Aldrich (St. Louis, MO, USA). Dulbecco’s Modified Eagle Medium (DMEM), 0.25% trypsin-EDTA solution, penicillin/streptomycin (P/S) solution, and fetal bovine serum (FBS) were purchased from Gibco (Grand Island, NY, USA). Dimethyl sulfoxide (DMSO) was purchased from Merck (Darmstadt, Germany). PGE_2_ ELISA assay kit was obtained from R&D Systems (Minneapolis, MN, USA). TNF-α ELISA assay kit (ab100747) and nitric oxide assay kit (ab65328) were purchased from Abcam (Waltham, MA, USA). The specific primers were obtained from Integrated DNA Technologies (Coralville, IA, USA). KAPA SYBR FAST One-step RT-qPCR kit was purchased from KAPA biosystems (Wilmington, MA, USA). RNeasy kit was obtained from Qiagen (Hilden, Germany).

### 3.2. Plant Materials and Isolated Lignans

The *M. sirikitiae* leaves were collected from Mae Surin Waterfall National Park, Mae Hong Sorn Province, Thailand (Latitude 18.94271; Longtitude 98.07122). A voucher specimen (BKF144972) of the plant has been deposited at the Forest Herbarium, Royal Forestry Department, Bangkok, Thailand. The methanol extract of *M. sirikitiae* leaves together with its lignans **1**–**6**, including (−)-epieudesmin (**1**), (−)-phylligenin (**2**), 2-(3,4-dimethoxyphenyl)-6-(3,5-dimethoxyphenyl)-3,7-dioxabicyclo[3.3.0]octane (**3**), magnone A (**4**), mitrephoran (**5**), 3′,4-*O*-dimethylcedrusin (**6**) were prepared as described in our previous study [10]. Briefly, 1 kg of ground dried *M. sirikitiae* leaves was extracted by maceration with methanol. After the solvent was evaporated, 102.0 g of methanol leaf extract was obtained. Solvent–solvent partitioning of the methanol extract (90.0 g) resulted in four subfractions, including hexane (20.7 g), ethyl acetate (21.1 g), butanol (47.6 g), and aqueous (24.2 g) subfractions. Separation of the mixture of hexane and ethyl acetate subfractions by column chromatography using silica gel P60 40–63 µm (SiliaFlash^®^; Silicycle) and Sephadex™ LH-20 (GE Healthcare Life Sciences) as stationary phases afforded six isolated lignans, **1**–**6**. The colorless crystals of (−)-epieudesmin (**1**) (197.5 mg), (−)-phylligenin (**2**) (717.4 mg), magnone A (**4**) (24.0 mg), and mitrephoran (**5**) (12.4 mg) were obtained after recrystallization from mixed solvent of methylene chloride and methanol. Moreover, 2-(3,4-dimethoxyphenyl)-6-(3,5-dimethoxyphenyl)-3,7-dioxabicyclo[3.3.0]octane (**3**) (9.6 mg) and 3′,4-*O*-dimethylcedrusin (**6**) (141.2 mg) were isolated as pale-yellow semisolids. The chemical structures of the isolated lignans were elucidated by their spectroscopic and physical characteristic data (see Supplementary Materials).

### 3.3. Cell Culture and Treatment

Murine macrophage RAW 264.7 cells obtained from American Type Culture Collection (ATCC, #TIB-71; Rockville, MD, USA) were cultured in DMEM plus 10% FBS and 1% P/S and incubated under a temperature 37 °C in a humidified atmosphere of 5% CO_2_ incubator as previously described [29]. The cells were grown in culture dishes. After the confluence of cells reached 80%, the cell density was calculated and used for the subsequent experiment. The cells were passaged by trypsinization every 3–5 days.

### 3.4. Determination of Cytotoxicity by MTT Assay

Cell viability was determined by MTT assay as previously described [30]. RAW 264.7 cells were seeded in 96-well culture plates at a 1 × 10^4^ cells/well density in DMEM supplemented with 1% FBS and 1% P/S and incubated in a humidified 37 °C, 5% CO_2_ incubator for 24 h. After incubation, the cultured cells were treated with various concentrations of the methanol extract of *M. sirikitiae* leaves and isolated lignans (0.05–250 µg/mL, diluted with DMEM), or dimethyl sulfoxide (DMSO; vehicle) and then incubated for 36 h. The culture medium was removed and added with MTT solution (1 mg/mL in DMEM). After incubated for 4 h, the MTT solution was removed before adding DMSO to dissolve the formed purple formazan crystals. Each experiment was performed in triplicate. The absorbance was detected using an Infinite M200 microplate reader (Tecan) at a wavelength of 570 nm. The results were shown in the graph of percentage of cell viability (% cell viability = [A_treated cells_/A_untreated cells_] × 100) against concentrations.

### 3.5. Enzyme-Linked Immunosorbent Assay (ELISA) for PGE_2_ and TNF-α Measurement

RAW 264.7 cells (2 × 10^5^ cells/well) were plated in 6-well culture plates overnight. In a serum-free medium, cells were pretreated with the methanol extract (1, 10 µg/mL), isolated lignans (5, 10 µg/mL), or indomethacin (10 µM; NSAID) for 3 h and subsequently treated with LPS (5 µg/mL) for 24 h. The supernatants were collected and stored at −80 °C until analysis. The assessment of PGE_2_ and TNF-α levels was quantified with the mouse PGE_2_ and TNF-α ELISA kits following the manufacturer’s instructions.

### 3.6. Measurement of Nitric Oxide Production

Nitrate and nitrite concentrations were assayed by Griess reagent using a nitric oxide assay kit as described previously [31]. RAW 264.7 cells (2 × 10^5^ cells/well) were plated in 6-well culture plates overnight. Under the serum-free condition, cells were pretreated with the methanol extract (1, 10 µg/mL), or isolated lignans (5, 10 µg/mL) for 3 h and subsequently treated with LPS (5 µg/mL) for 24 h. The absorbance was measured at a wavelength of 540 nm using the Infinite M200 microplate reader (TECAN). Nitrate and nitrite concentrations in the samples were calculated from the standard curve following the manufacturer’s instructions. The amounts of nitrate and nitrite accurately reflect the NO production in RAW 264.7 cells.

### 3.7. Measurement of mRNA Expression of Inflammatory Biomarkers

The effects of the methanol extract and the isolated lignans on the synthesis of inflammatory biomarkers were determined by measurement of mRNA expression. Under serum-free condition, RAW 264.7 cells (2 × 10^5^ cells/well) were treated with the methanol extract (1, 10 µg/mL), or isolated lignans (5, 10 µg/mL) for 6 h. The total mRNA of RAW 264.7 cells was isolated with RNeasy kit (Qiagen). The mRNA expression of inflammatory biomarkers was determined by using the KAPA SYBR FAST One-step RT-qPCR kit with gene-specific primer sequences (Table 1) and Mx 3005p Real-Time PCR system (Stratagene) as previously described [32]. The mRNA expression level of inflammatory biomarkers was calculated by the comparative cycle threshold (CT) method. Glyceraldehyde-3-phosphate dehydrogenase (GAPDH) was used as the housekeeping gene.

### 3.8. Statistical Analysis

Data are presented as the mean ± SEM from 3–5 independent experiments. Statistical analysis was obtained by using SPSS software (version 25). The difference between groups was evaluated using one-way ANOVA with a post hoc test. The value of *p*-value less than 0.05 (*p* < 0.05) was accepted significant.

## 4. Conclusions

This study revealed that the methanol extract of *M. sirikitiae* leaves and isolated lignans, including (−)-phylligenin (**2**) and 3′,4-*O*-dimethylcedrusin (**6**) possess the anti-inflammatory activity by suppressing LPS-induced iNOS and COX-2 synthesis which contribute to a reduction in PGE_2_ and nitric oxide secretions in RAW 264.7 macrophages. Moreover, 2-(3,4-dimethoxyphenyl)-6-(3,5-dimethoxyphenyl)-3,7-dioxabicyclo[3.3.0] octane (**3**) and mitrephoran (**5**) elicit the anti-inflammatory effects through inhibition of LPS-induced the secretion and synthesis of TNF-α in RAW 264.7 cells. Hence, these findings suggested that several lignans isolated from *M. sirikitiae* leaves might be potential therapeutic candidates for the inhibition and prevention of various inflammatory diseases.

## Figures and Tables

**Table 1:** The gene-specific primers for RT-qPCR (mouse).

Gene-Specific Primers		Sequences
COX-2	Sense	5′-tgcatgtggctgtggatgtcatcaa-3′
	Antisense	5′-cactaagacagacccgtc atctcca-3′
IL-6	Sense	5′-gacaaagccagagtccttcagagag-3′
	Antisense	5′-ctaggtttgccgagtagatctc-3′
IL-10	Sense	5′-gctggacaacatactgctaacc-3′
	Antisense	5′-atttccgataaggcttggcaa-3′
iNOS	Sense	5′-gtgttccaccaggagatgttg-3′
	Antisense	5′-ctcctgcccactgagttcgtc-3′
NF-κB	Sense	5′-gaaattcctgatccagacaaaaac-3′
	Antisense	5′-atcacttcaatggcctctgtgtag-3′
TNF-α	Sense	5′-atgagcacagaaagcatgatc-3′
	Antisense	5′-tacaggcttgtcactcgaatt-3′
GAPDH	Sense	5′-gcctgcttcaccaccttc-3′
	Antisense	5′-ggctctccagaacatcatcc-3′

## Data Availability

The data presented in this study are available in supplementary material.

## Supplementary Materials

The following supporting information can be downloaded at: https://www.mdpi.com/article/10.3390/molecules27103313/s1. Spectroscopic and physical data of isolated lignans 1–6 from *Mitrephora sirikitiae* Leaf extract.
