# Supplementary material for: Anti-Inflammatory Effects of Mitrephora sirikitiae Leaf Extract and Isolated Lignans in RAW 264.7 Cells
Source: Molecules. 2022 May 21;27(10):3313. doi: 10.3390/molecules27103313 (PMC9147141; doi:10.3390/molecules27103313)
Supplement: Supplementary file 1 [file molecules-27-03313-s001.zip › molecules-1717888-supplementary.pdf]

Supplementary Materials

Anti-inflammatory effects of *Mitrephora sirikitiae* leaf extract and isolated lignans in RAW 264.7 cells

Supachoke Mangmool [1], Chayaporn Limpichai [2], Khine Kyi Han [3], Vichai Reutrakul [4,5] and Natthinee Anantachoke [2,5,*]

[1] Department of Pharmacology, Faculty of Science, Mahidol University, Bangkok 10400, Thailand; supachoke.man@mahidol.ac.th (S.M.)

[2] Department of Pharmacognosy, Faculty of Pharmacy, Mahidol University, Bangkok 10400, Thailand;
chayapornlim@gmail.com (C.L.)

[3] Department of Pharmacology, University of Pharmacy, Yangon, 11031, Myanmar; khinekyihanuop@ gmail.com (K.K.H.)

[4] Department of Chemistry, Faculty of Science, Mahidol University, Bangkok 10400, Thailand
vichai.reu@mahidol.ac.th (V.R.)

[5] Center of Excellence for Innovation in Chemistry, Faculty of Science, Mahidol University, Bangkok 10400, Thailand; natthinee.ana@mahidol.ac.th (N.A.)

* Correspondence: natthinee.ana@mahidol.ac.th

Spectroscopic and physical data of isolated lignans 1-6 from *Mitrephora sirikitiae* Leaf extract.

1. (–)-Epieudesmin (1)

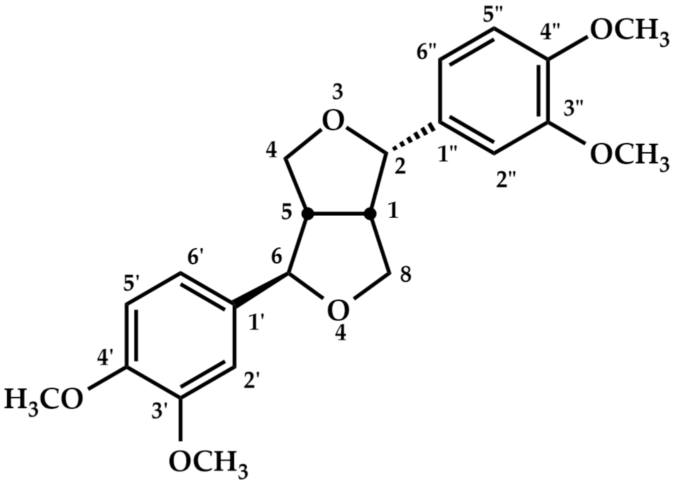

Physical characteristics: Colorless crystals from CH_2Cl_2/MeOH; m.p. 128-130 ^{o}C

$[\alpha]_D^{25}$: -123.66 (*c* 1.0, $CHCl_3$)

UV λ_{max}^{MeOH} nm (log ε): 231 (3.91), 279 (4.38)

FTIR (neat) ν_{max}: 2918, 2915, 1607, 1590, 1463, 1265, 1160, 1142 cm^{-1}

HR-ESI-MS ***m/z***: 409.1621 $[M+Na]^+$

EI-MS *m/z* (% relative intensity): 386 $[M]^+$ (68), 355 $[M-OCH_3]^+$ (6), 340 $[M-OCH_3-CH_3]^+$ (9), 309 $[M-2OCH_3-CH_3]^+$ (7), 220 (10), 205 (13), 194 (14), 189 (15), 177 (36), 165 (100), 151 (26)

^{1}H-NMR (500 MHz, $CDCl_3$) δ_H *ppm* (mult., *J* (Hz)): 6.97 (1H, *d*, 1.1 Hz, H-2″), 6.95 (1H, *d*, 1.9 Hz, H-2′), 6.93 (1H, *dd*, 8.2, 1.9 Hz, H-6′), 6.90 (1H, *d*, 8.4 Hz, H-6″), 6.88 (1H, *d*, 8.4 Hz, H-5″), 6.87 (1H, *d*, 8.2 Hz, H-5′), 4.91 (1H, *d*, 5.7 Hz, H-2), 4.48 (1H, *d*, 7.2 Hz, H-6), 4.16 (1H, *dd*, 9.4, 0.8 Hz, H-4), 3.94 (3H, *s*, OMe-3″), 3.93 (3H, *s*, OMe-3′), 3.92 (3H, *s*, OMe-4″), 3.91 (3H, *s*, OMe-4′), 3.88 (1H, *m*, H-8), 3.88 (1H, *m*, H-4), 3.39 (1H, *m*, H-1), 3.34 (1H, *t*, 8.4 Hz, H-8), 2.95 (1H, *m*, H-5)

^{13}C-NMR (125 MHz, $CDCl_3$) δ_C *ppm*: 149.28 (C-3′), 148.90 (C-3″), 148.77 (C-4′), 148.07 (C-4″), 133.72 (C-1′), 131.00 (C-1″), 118.47 (C-6′), 117.75 (C-6″), 111.10 (C-5',C-5″), 109.23 (C-2′), 109.01 (C-2″), 87.64 (C-6), 82.07 (C-2), 71.04 (C-4), 69.74 (C-8), 55.97 (OMe-4′), 55.95 (OMe-4″), 55.93 (OMe-3′, OMe-3″), 54.50 (C-5), 50.18 (C-1)

2. (–)-Phylligenin (2)

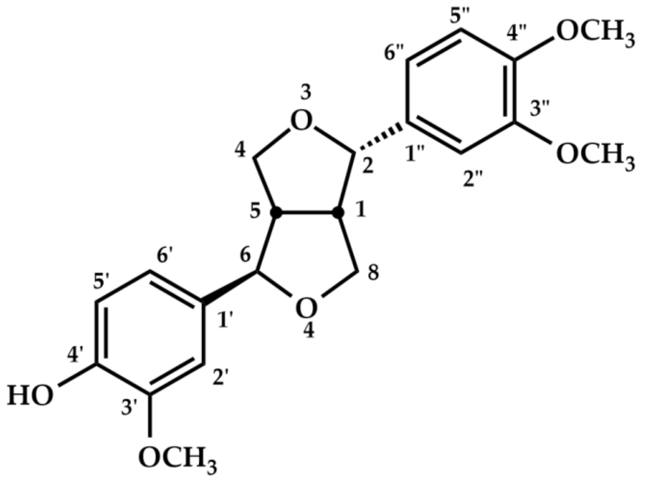

Physical characteristics: Colorless crystals from CH_2Cl_2/MeOH; m.p. 135-136 °C;

$[\alpha]_D^{24}$:-128.53 (*c* 1.0, $CHCl_3$)

UV λ_{max}^{MeOH} nm (log ε): 231 (3.95), 280 (4.38)

FTIR (neat) ν_{max}: 3430, 2961, 2939, 2844, 1607, 1589, 1515, 1469, 1460, 1447, 1460, 1417, 1382, 1347, 1278, 1264, 1256, 1236, 1193, 1157, 1143, 1127, 1076, 1023 cm^{-1}

HR-ESI-MS *m*/*z*: 395.1471 $[M+Na]^+$

EI-MS *m*/*z* (% relative intensity): 372 $[M]^+$ (68), 341 $[M-OCH_3]^+$ (6), 326 $[M-OCH_3-CH_3]^+$ (9), 273 (8), 205 (21), 194 (13), 189 (19), 177 (27), 165 (42), 151 (100)

^{1}H-NMR (500 MHz, $CDCl_3$) δ_H *ppm* (mult., *J* (Hz)): 6.99 (1H, *br s*, H-2′), δ 6.96 (1H, *d*, 1.7 Hz, H-2″), 6.91 (2H, *m*, H-6′, H-6″), 6.93 (2H, *d*, 8.1 Hz, H-5′, H-5″), 5.74 (1H, *s*, OH-4′), 4.92 (1H, *d*, 5.5 Hz, H-6), 4.48 (1H, *d*, 7.1 Hz, H-2), 4.18 (1H, *d*, 9.7 Hz, H-8), 3.99 (3H, *s*, OMe-3″), 3.93 (3H, *s*, OMe-3′), 3.92 (3H, *s*, OMe-4″), 3.89 (2H, *m*, H-4, H-8), 3.39 (2H, *m*, H-4, H-5), 2.95 (1H, *br dd*, 14.4, 7.2 Hz, H-1)

^{13}C-NMR (125 MHz, $CDCl_3$) δ_C *ppm*: 148.82 (C-3′), 146.77 (C-3″), 147.98 (C-4′), 145.36 (C-4″), 133.00 (C-1″), 130.95 (C-1′), 119.22 (C-6″), 117.72 (C-6′), 114.28 (C-5″), 111.00 (C-5′), 108.91 (C-2′), 108.56 (C-2″),

87.77 (C-2), 82.66 (C-6), 71.02 (C-8), 69.73 (C-4), 55.95 (OMe-4″), 55.93 (OMe-3′, OMe-3″), 54.52 (C-1), 50.16 (C-6)

3. 2-(3,4-Dimethoxyphenyl)-6-(3,5-dimethoxyphenyl)-3,7-dioxabicyclo[3.3.0]octane (3)

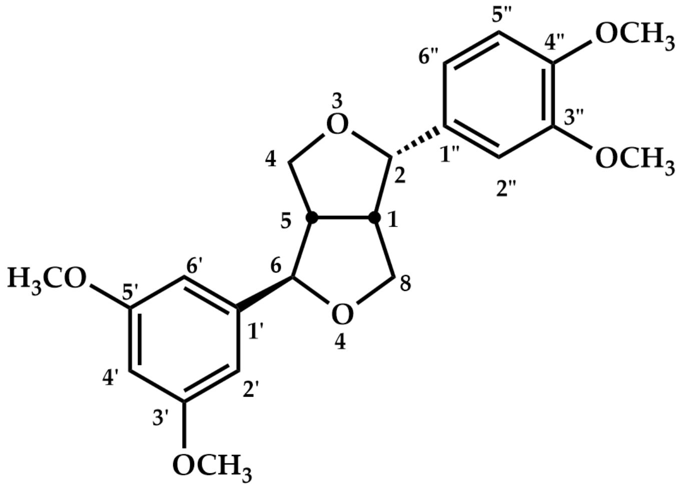

Physical characteristics: A pale yellow semisolid

$[\alpha]_D^{28.7}$: -17.68 (*c* 1.0, MeOH)

UV λ_{max}^{MeOH} nm (log ε): 230 (4.30), 280 (3.91)

FTIR (neat) ν_{max}: 2930, 2850, 1593, 1512, 1461, 1419, 1258, 1232, 1156, 1136, 1061, 1021 cm^{-1}

HR-ESI-MS *m*/*z*: 409.1621 $[M+Na]^+$

EI-MS *m*/*z* (% relative intensity): 386 $[M]^+$ (5), 384 $[M-2H]^+$ (34), 367 (14), 337 (41), 325 (82), 292 (32), 275 (20), 263 (27), 220 (32), 202 (41), 189 (100), 165 (85).

^{1}H-NMR (400 MHz, CD_3OD) δ_H *ppm* (mult., *J* (Hz)): *δ* 6.96 (1H, *s*, H-2′), 6.91 (3H, *br s*, H-4′, H-6′, H-2″), 6.88 (1H, *d*, 8.2 Hz, H-5″), 6.82 (1H, *dd*, 8.2, 1.7 Hz, H-6″), 4.64 (1H, *d*, 7.4 Hz, H-6), 4.50 (1H, *d*, 8.4 Hz, H-2), 4.25 (1H, *dd*, 8.8, 4.4 Hz, H-4), 3.95 (1H, *dd*, 8.8, 7.6 Hz, H-4), 3.82 (3H, *s*, 5′-OMe), 3.81 (3H, *s*, 3′-OMe), 3.79 (6H, *s*, 3″-OMe, 4″-OMe), 3.30 (1H, *m*, H-8), 3.23 (1H, *dd*, 16.5, 5.9 Hz, H-8), 2.54 (1H, *m*, H-5), 1.88 (1H, *m*, H-1)

^{13}C-NMR (100 MHz, CD_3OD) δ_C *ppm*: 150.51 (C-5′), 150.46 (C-3''), 149.98 (C-3′, C-4″), 137.49 (C-1″), 136.25 (C-1′), 120.61 (C-6″), 120.02 (C-6′), 112.85 (C-4′), 112.71 (C-5″), 111.73 (C-2″), 111.32 (C-2′), 84.83 (C-6), 73.36 (C-2), 71.43 (C-4), 62.69 (C-8), 56.57 (3″-OMe), 56.50 (3′-OMe, 5′-OMe), 56.44 (4″-OMe), 53.57 (C-1), 50.77 (C-5)

4. Magnone A (4)

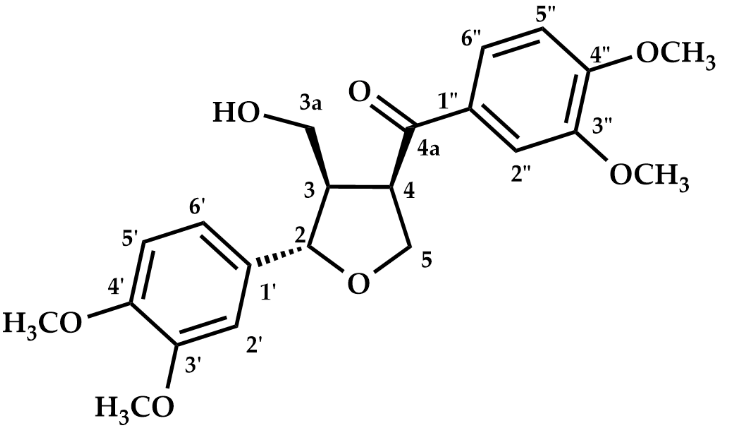

Physical characteristics: Colorless crystals from CH_2Cl_2/MeOH; m.p. 139-140 °C

$[\alpha]_D^{25}$: 18.54 (*c* 1.0, MeOH)

UV λ_{max}^{MeOH} nm (log ε): 230 (4.37), 276 (4.13), 305 (3.93)

FTIR (neat) ν_{max}: 3461, 2912, 2839, 1651, 1592, 1583, 1517, 1467, 1441, 1420, 1334, 1288, 1237, 1203, 1192, 1154, 1104, 1080, 1020 cm^{-1}

HR-ESI-MS *m/z*: 425.1584 $[M+Na]^+$

EI-MS *m/z* (% relative intensity): 368 (68), 355 (6), 340 (9), 309 (7), 220 (10), 205 (13), 194 (14), 189 (15), 177 (36), 165 (100), 151 (26).

^{1}H-NMR (400 MHz, $CDCl_3$) δ_H *ppm* (mult., *J* (Hz)): 7.66 (1H, *dd*, 8.2, 2.0 Hz, H-6″), *δ* 7.63 (1H, *d*, 2.0 Hz, H-2″), 7.09 (1H, *d*, 1.9 Hz, H-2′), 6.99 (1H, *dd*, 8.2, 1.9 Hz, H-6′), 6.98 (1H, *d*, 8.3 Hz, H-5″), 6.89 (1H, *d*, 8.2 Hz, H-5′), 4.75 (1H, *d*, 9.0 Hz, H-2), 4.37 (1H, *dd*, 11.2, 10.9 Hz, H-5), 4.24 (2H, *m*, H-4, H-5), 4.01 (3H, *s*, OMe-4″), 4.00 (3H, *s*, OMe-4′), 3.97 (3H, *s*, OMe-3″), 3.93 (3H, *s*, OMe-3′), 3.84 (2H, *dd*, 10.9, 4.5 Hz, H-3a), 2.77 (1H, *m*, H-3)

^{13}C-NMR (100 MHz, $CDCl_3$) δ_C *ppm*: 197.97 (C-4a), 153.70 (C-4″), 149.27 (C-4′, C-3″), 148.95 (C-3′), 132.94 (C-1′), 129.75 (C-1″), 123.23 (C-6″), 119.36 (C-6′, C-2″), 110.82 (C-5′), 110.72 (C-5″), 109.56 (C-2′), 83.82 (C-2), 70.90 (C-5), 61.51 (C-3a), 56.81 (OMe-4″), 56.06 (OMe-4′), 55.95 (OMe-3′, OMe-3″), 52.14 (C-3), 49.72 (C-4)

5. Mitrephoran (5)

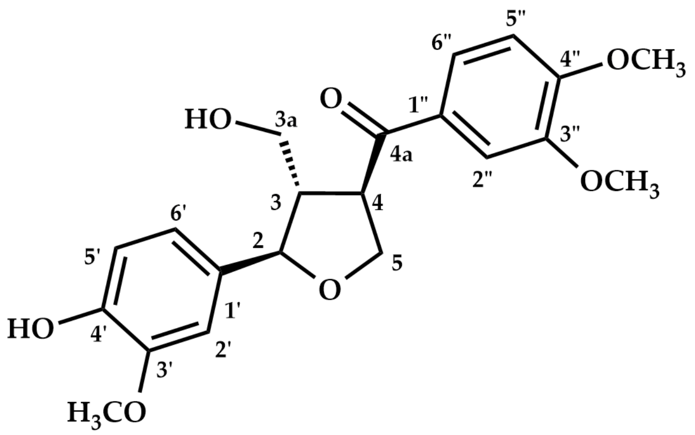

Physical characteristics: Colorless crystals were obtained from CH_2Cl_2/MeOH; m.p. 146-148 °C

$[\alpha]_D^{25}$**:** 14.00 (*c* 0.45, $CHCl_3$)

UV λ_{max}^{MeOH} **nm (log ε)**: 230 (4.37), 276 (4.13), 305 (3.93)

FTIR (neat) ν_{max}: 3396, 3259, 2954, 2872, 2841, 1672, 1597, 1587, 1515, 1463, 1439, 1418, 1357, 1347, 1287, 1259, 1158, 1122, 1088, 1060, 1033, 1020, 1005, and 968 cm^{-1}

HR-ESI-MS ***m/z***: 411.1420 $[M+Na]^+$

EI-MS ***m/z*** **(% relative intensity)**: 388 $[M]^+$ (8), 218 (14), 196 (38), 180 (93), 165 (100), and 151 (60)

^{1}H-NMR (400 MHz, $CDCl_3$) δ_H ***ppm*** **(mult.,** ***J*** **(Hz))**: 7.67 (1H, dd, 8.4, 2.0 Hz, H-6″), 7.63 (1H, d, 2.0 Hz, H-2″), 7.08 (1H, br s, H-2′), 6.98 (1H, d, 8.4 Hz, H-5″), 6.94 (1H, m, H-6′)*, 6.93 (1H, m, H-5′)*, 4.73 (1H, d, 9.1 Hz, H-2), 4.37 (1H, m, H-5), 4.24 (2H, m, H-4, H-5), 4.02 (3H, s, 4″-OMe), 4.01 (3H, s, 3″-OMe), 3.99 (3H, s, 3′-OMe), 3.83 (1H, dd, 10.9, 4.4 Hz, H-3a), 3.73 (1H, dd, 10.9, 5.6 Hz, H-3a), 2.96 (1H, m, H-3)

* H-5' and H-6' were observed as broad singlets because of the virtual coupling effects.

^{13}C-NMR (100 MHz, $CDCl_3$) δ_C ***ppm***: 198.04 (C-4a), 153.70 (C-4″), 149.26 (C-3″), 146.88 (C-3′), 145.63 (C-4′), 132.31 (C-1′), 129.77 (C-1″), 123.23 (C-6″), 120.17 (C-6′), 114.04 (C-5′), 110.57 (C-2″), 110.12 (C-5″), 108.92 (C-2′), 83.91 (C-2), 70.85 (C-5), 61.44 (C-3a), 56.18 (4″-OMe), 56.06 (3″-OMe), 56.02 (3′-OMe), 52.21 (C-3), 49.67 (C-4)

6. 3′,4-*O*-Dimethylcedrusin (6)

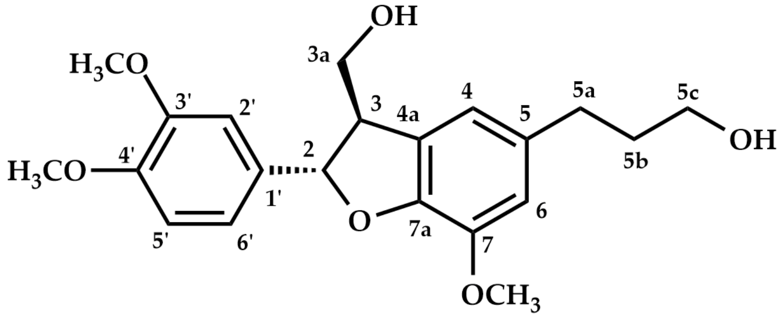

Physical characteristics: A pale yellow semisolid; $[\alpha]_D^{28.7}$ -8.52 (*c* 0.1, $CHCl_3$)

UV λ_{max}^{MeOH} nm (log ε): 231 (4.19), 281 (3.73)

FTIR (neat) ν_{max}: 3375, 2934, 1604, 1515, 1497, 1451, 1422, 1323, 1259, 1235, 1210, 1137, 1021 cm^{-1}

HR-ESI-MS *m/z*: 397.1624 $[M+Na]^+$

EI-MS *m/z* (% relative intensity): 374 $[M]^+$ (4), 356 $[M-H_2O]^+$ (83), 341 $[M-CH_3-H_2O]^+$ (100), 325 $[M-OCH_3-H_2O]^+$ (39), 309 (15).

^{1}H-NMR (400 MHz, CD_3OD) δ_H *ppm* (mult., *J* (Hz)): 6.91 (1H, *br s*, H-2′), 6.88 (1H, *d*, 8.2, H-5′), 6.82 (1H, dd, 8.2, 1.7, H-6′), 6.72 (1H, *s*, H-6), 6.71 (1H, *s*, H-4), 5.52 (1H, *d*, 6.1 Hz, H-2), 3.84 (3H, *s*, 7-OMe), 3.81 (1H, *dd*, 11.0, 5.8 Hz, Hb-3a), 3.79 (3H, *s*, 4′-OMe), 3.77 (3H, *s*, 3′-OMe), 3.74 (1H, *dd*, 11.0, 7.3 Hz, Ha-3a), 3.56 (2H, *t*, 6.5 Hz, H-5c), 3.45 (1H, *m*, H-3), 2.61 (2H, *t*, 7.7 Hz, H-5a), 1.80 (2H, *tt*, 7.7, 6.5 Hz, H-5b)

^{13}C-NMR (100 MHz, CD_3OD) δ_C *ppm*: 150.56 (C-3′), 150.25 (C-4′), 147.50 (C-7a), 145.22 (C-7), 136.99 (C-5), 136.19 (C-1′), 129.73 (C-4a), 119.43 (C-6′), 117.92 (C-4), 114.11 (C-6), 112.87 (C-5′), 110.70 (C-2′), 88.70 (C-2), 65.02 (C-3a), 62.23 (C-5c), 56.74 (7-OMe), 56.49 (4′-OMe), 56.42 (3′-OMe), 55.50 (C-3), 35.80 (C-5b), 32.89 (C-5a)
